# An Eighteen Serum Cytokine Signature for Discriminating Glioma from Normal Healthy Individuals

**DOI:** 10.1371/journal.pone.0137524

**Published:** 2015-09-21

**Authors:** Mamatha B. Nijaguna, Vikas Patil, Alangar S. Hegde, Bangalore A. Chandramouli, Arimappamagan Arivazhagan, Vani Santosh, Kumaravel Somasundaram

**Affiliations:** 1 Department of Microbiology and Cell Biology, Indian Institute of Science, Bangalore 560012, India; 2 Department of Neurosurgery, Sri Satya Sai Institute of Higher Medical Sciences, Bangalore 560066, India; 3 Department of Neurosurgery, National Institute of Mental Health and Neuro Sciences, Bangalore 560029, India; 4 Department of Neuropathology, National Institute of Mental Health and Neuro Sciences, Bangalore 560029, India; University of Alabama at Birmingham, UNITED STATES

## Abstract

Glioblastomas (GBM) are largely incurable as they diffusely infiltrate adjacent brain tissues and are difficult to diagnose at early stages. Biomarkers derived from serum, which can be obtained by minimally invasive procedures, may help in early diagnosis, prognosis and treatment monitoring. To develop a serum cytokine signature, we profiled 48 cytokines in sera derived from normal healthy individuals (n = 26) and different grades of glioma patients (n = 194). We divided the normal and grade IV glioma/GBM serum samples randomly into equal sized training and test sets. In the training set, the *P*rediction *A*nalysis for *M*icroarrays (PAM) identified a panel of 18 cytokines that could discriminate GBM sera from normal sera with maximum accuracy (95.40%) and minimum error (4.60%). The 18-cytokine signature obtained in the training set discriminated GBM sera from normal sera in the test set as well (accuracy 96.55%; error 3.45%). Interestingly, the 18-cytokine signature also differentiated grade II/Diffuse Astrocytoma (DA) and grade III/Anaplastic Astrocytoma (AA) sera from normal sera very efficiently (DA vs. normal–accuracy 96.00%, error 4.00%; AA vs. normal–accuracy 95.83%, error 4.17%). *K*yoto *E*ncyclopedia of *G*enes and *G*enomes (KEGG) pathway analysis using 18 cytokines resulted in the enrichment of two pathways, cytokine-cytokine receptor interaction and JAK-STAT pathways with high significance. Thus our study identified an 18-cytokine signature for distinguishing glioma sera from normal healthy individual sera and also demonstrated the importance of their differential abundance in glioma biology.

## Introduction

Glioblastoma (GBM), a grade IV glioma, is the most common and malignant adult primary brain tumor with a poor survival [1,2]. Despite advances in treatment strategies, the prognosis is only marginally improved, which prompts for further understanding of the disease pathogenesis and biological behavior [3]. The clinical features of raised intracranial pressure, onset of a neurological deficit or late onset seizure raises a possibility of a brain tumor in a patient. Imaging of brain, either by ***C***omputerized ***T***omography (CT) scan or ***M***agnetic ***R***esonance ***I***maging (MRI), is the primary modality of making a possible diagnosis of glioma. However, a number of differential diagnoses can be considered for a parenchymal brain lesion seen in an MRI, one of which is glioma. Therefore, a biopsy of the lesion is mandatory to obtain a histological diagnosis, which is the gold standard for the diagnosis and management. A number of efforts have been made to differentiate various grades of glioma using various radiological techniques earlier [4,5,6,7,8]. Similarly, radiological features to differentiate glioma from other brain parenchymal lesions have also been described [9,10]. Despite these findings, histological diagnosis is mandatory, as the accuracy of the radiological features are not sufficient and cannot be considered for making clinical management decisions. These indicate a clear need for accurate and robust biomarkers which could assist in diagnosis and grading. Several studies have identified molecular signatures in GBM tumors, based on gene expression, miRNA expression or methylation profile which can classify and predict prognosis [11,12,13,14,15,16,17,18]. In contrast to these markers, the use of serum-based biomarkers has several advantages. Serum can be obtained by less invasive method, tests can be repeated many times during the course of the disease and serum biomarkers may reflect the systemic condition of an individual at any given time. Moreover, the serum contains variety of macromolecules like DNA, RNA, miRNA and proteins derived from the tumor which could serve as potential serum biomarkers. An availability of serum based biomarker can be used to monitor the response to therapy, recurrence of the disease and possibly make initial diagnosis. The identification of early tumor recurrence and assessment of tumor response will lead to better clinical management. While single molecule based serum biomarkers are reported for glioma diagnosis [19,20,21], a multi-marker panel with robust classification accuracy will most likely be more appropriate, in the background of multifactorial origin and heterogeneous nature of the disease.

Cytokines play a major role in infiltration of immune cells and their function. Although the central nervous system is considered to be an immune-privileged site, numerous immune cell infiltrations have been reported in GBM specimens. Among them, macrophages/microglial cells which are present in abundance represent almost one-third of the tumor mass and show grade-dependent increase [22,23]. Several reports suggest an important role of cytokines in cancer development and their implication in cancer therapy [24]. In GBM, altered cytokine levels in serum and transcript expression has been reported [25,26]. Therefore, we hypothesized that the perturbed levels of cytokines are responsible for immune cell infiltration in case of glioma and these altered levels may be reflected in serum of glioma patients as well.

In the current study with an aim to develop a serum cytokine signature for glioma diagnosis, we profiled 48 cytokines using sera of patients with glioma and normal healthy controls. ***P***rediction ***A***nalysis for ***M***icroarrays (PAM) identified an 18-cytokine signature which distinguished glioma serum samples- grade IV/Glioblastoma (GBM), grade II/Diffuse Astrocytoma (DA) and grade III/Anaplastic Astrocytoma (AA) from normal serum samples with high accuracy. Pathway analysis identified a strong association between these 18 cytokines and glioma activated pathways which are suggestive of the importance of the identified cytokines in glioma biology.

## Materials and Methods

### Serum samples and clinical data

Blood samples were collected from patients who were diagnosed with glioma (DA, AA and GBM) and managed by surgical decompression at National Institute of Mental Health and Neurosciences (NIMHANS) and Sri Satya Sai Institute of Higher Medical Sciences (SSSIHMS), Bangalore, India. This study has been approved by the ethics committee of NIMHANS and SSSIHMS and patient’s written consent was obtained. All the serum samples from glioma patients were collected prior to surgery. The histology of all the operated tumors was reviewed by an experienced neuropathologist and diagnosis was made. The grading of glioma was done as per WHO 2007 classification criteria [27]. Normal blood samples were collected from healthy individuals at Indian Institute of Science (IISc) Bangalore, India with prior consent. Normal samples used in this study are age and gender matched with GBM samples but only gender matched with DA and AA since the lower grades are generally of younger age group (**S1 Fig**). The blood samples from glioma patients and normal controls were allowed to clot at 4°C overnight followed by centrifugation at 4°C for 5 min at 1000 rpm to separate serum (upper phase) from clot. Serum samples were stored at -80°C until use.

Among the GBM samples used in this study, a subset of 96 samples was prospectively recruited. The clinical and follow-up information for these patients is given in **S1 Table**. All these patients were adults and underwent total/near total excision of the tumor. These patients exhibited post-operative Karnofsky’s Performance Status (KPS) ≥ 70. After surgery, all patients were treated with standard therapy, which included radiotherapy (total dose of 60 Gy, given in 30 fractions over 6 weeks) with concomitant temozolomide (TMZ) chemotherapy (100 mg/day for 45 days), followed by five cycles of TMZ treatment at a dose of 150 mg/sq.m body surface area. These patients were followed up at regular intervals and evaluated clinically and radiologically by MRI. Overall survival was defined as the time between surgery and patient death due to disease.

### Serum cytokine profiling

Serum cytokine profiling was done using serum samples from normal (n = 26), GBM (n = 148), DA (n = 24) and AA (n = 22) by bead array method. We used commercially available human cytokine kits: 21-plex and 27-plex (Bio-rad) and followed the protocol according to manufacturer’s instructions (**S2 Table**). The basic principle is based on sandwich ELISA. In this assay, serum samples were diluted 4 times with dilution buffer and incubated with beads containing specific antibodies directed against different cytokines. After thorough washing step, a biotinylated detection antibody was added. Next, a streptavidin-phycoerythrin reporter complex was added and incubated for 15 mins. The plate was washed and read using bio-plex system, a dual-laser flow-based microplate reader. The lasers and associated optics detect the internal fluorescence of the individual bead as well as the fluorescent reporter signal on the bead surface. The standard curve was generated using known concentrations of purified cytokines which later allowed the quantification (pico gram /mL) of each cytokine in the serum samples.

### Prediction Analysis for Microarrays

The ***P***rediction ***A***nalysis for ***M***icroarrays (PAM) was carried out using the PAM package in the R software (version 3.1.0). In PAM, the nearest shrunken centroids method was used to identify a subgroup of genes that best characterizes a predefined class [28]. The prediction accuracy was estimated by 10-fold cross-validation. The data set was divided into 10 subsets of equal size. Nine subsets were used as training and one subset was used as test. This process was repeated 10 times. Nearest shrunken centroid classification method “shrinks” each of the class centroids towards the overall centroid for all by threshold. The user would choose threshold value for achieving minimum error and minimum number of genes.

### Principal Component Analysis

In ***P***rincipal ***C***omponent ***A***nalysis (PCA), orthogonal transformation is used to convert a set of variables into a set of values of linearly uncorrelated variables called principal components. The number of principal components is less than or equal to the number of original variables. The first two or three components account for the largest possible variance in the dataset. PCA was performed by using R software (version 3.1.0).

### Support Vector Machine

For internal validation, we have used ***S***upport ***V***ector ***M***achine (SVM). Many prediction methods use SVM for classification of dataset into two or more classes. For a given set of binary classes training examples, SVM can map the input space into higher dimensional space and seek a hyperplane to separate the positive data examples from the negative ones with the largest margin. GBM and normal samples were divided randomly into five sub-groups containing equal number of the respective samples. Subsequently, one sub-group of normal and GBM was considered as a test set, while the remaining four sub-groups were considered as training set. This exercise was repeated keeping each of the sub-groups as test while the other four sub-groups as training set. In this way, SVM models were built five times to give five folds, wherein every sub-group was considered as a test set and the remaining sub-groups as training set in each fold. The accuracy for each fold in the training set and testing set was calculated and finally the overall accuracy of all five folds is given.

### Random Subset Sampling

The predictive accuracy of 18 cytokines was also analyzed in a subset of 44 samples (50% of the training set; GBM = 37, normal = 7) by random subset sampling. PAM was used to predict accuracy of 18 cytokines in the random subset sampling.

### Network analysis and other statistical analysis

***K***yoto ***E***ncyclopedia of ***G***enes and ***G***enomes (KEGG) pathway analysis was carried out using The ***D***atabase for ***A***nnotation, ***V***isualization and ***I***ntegrated ***D***iscovery (DAVID) Bioinformatics Resources tool version 6.7. A non-parametric t-test was performed to find out the significance between two groups and one-way ANOVA was performed to analyze more than one group using Graph Pad Prism 5.01. The prognostic significance of serum levels of 18 cytokines was tested by univariate and multivariate Cox proportional hazard analysis using SPSS software version 19. ***R***eceiver ***O***perating ***C***haracteristic (ROC) curves were plotted using sensitivities and specificities calculated from all possible cutoff values of serum levels of 18 cytokines using statistical analysis software MedCalc. A p value less than 0.05 were considered significant for all analyses. Supervised hierarchical clustering of significantly differentially expressed cytokines was carried out using the MultiExperiment Viewer (MeV).

## Results

### Serum cytokine signature-overall workflow

**Fig 1:**
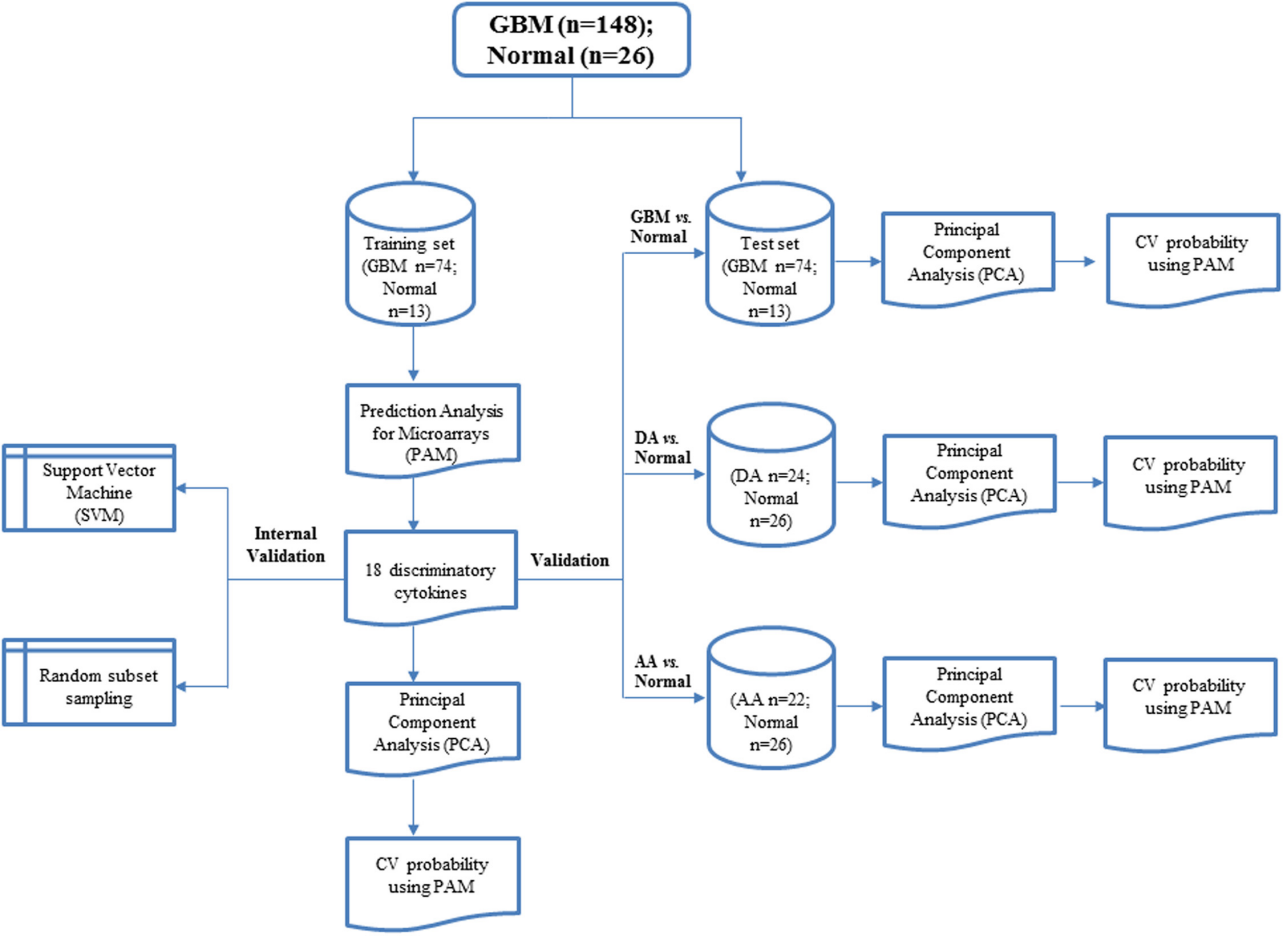
Schematic representation of the work flow of statistical analysis to derive at serum cytokine signature. The total dataset, consisting of serum levels of 48 cytokines for all normal (n = 26) and GBM (n = 148) samples was randomly divided into two equal halves as training and test set. The training set was subjected to PAM analysis and 18 discriminatory cytokines were identified. This was further validated by PCA and cross validated probability by PAM. Internal validation using SVM and random subset sampling were carried out in the training set. Subsequently, the 18-cytokine signature was validated in the test set as well as in DA *vs*. normal and AA *vs*. normal.

We profiled 48 cytokines (**S2 Table)** in the sera derived from normal healthy individuals (n = 26), DA (n = 24), AA (n = 22) and GBM (n = 148) patients using a bead array based platform. The clinical details of the patient cohort used in this study are given in the **S1 Table**. The cytokine levels were log2 transformed before using for further analysis. To identify a serum cytokine signature which can distinguish GBM serum from normal healthy individual serum, the levels of 48 cytokines in the sera derived from 148 GBM patients and 26 normal healthy individuals was subjected to ***P***rediction ***A***nalysis for ***M***icroarrays (PAM). The schematic of the analysis workflow is shown (**Fig 1**). First, we randomly divided our cohort into training set and test set, each having equal proportion of normal and GBMs. Next, the training set (normal; n = 13 and GBM; n = 74) was analyzed by PAM to identify a subset of cytokines that can distinguish normal from GBM. PAM analysis identified an 18-cytokine signature which was further validated through ***P***rincipal ***C***omponent ***A***nalysis (PCA) and cross validated probability in the training set as well as in test set. The ability of the 18-cytokine signature to distinguish DA and AA from normal sera was also pursued.

### Identification of 18-cytokine signature to distinguish GBM sera from normal sera in the training set

**Fig 2:**
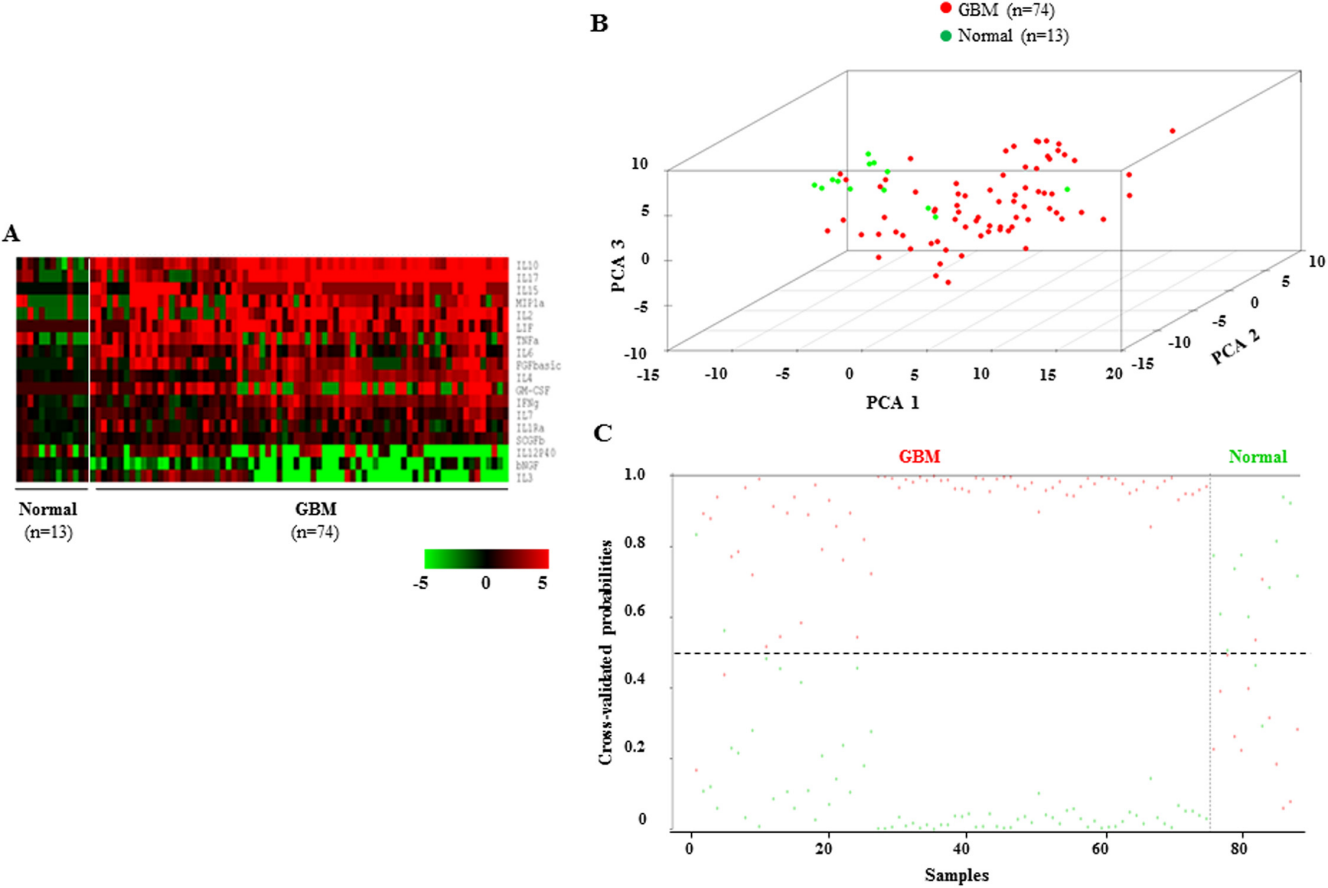
Serum cytokine levels, PCA and cross validated probabilities in training set. **A.** Heat map of supervised one-way hierarchical clustering of 18 PAM-identified cytokines in normal and GBM sera of the training set. A dual-color code was used, with red and green indicating high and low abundance, respectively. The white line separates normal from GBM samples. **B.** PCA was performed using serum levels of 18 PAM-identified cytokines of normal and GBM sera in the training set. A scatter plot was generated using first three principal components for each sample. The color code of the samples is as indicated. **C.** The graph shows detailed probabilities of 10-fold cross-validation for the samples of training set based on the serum levels of 18 PAM-identified cytokines. The probability of a given sample as normal (green color) and GBM (red color) are shown. This was predicted by the PAM program, based on which type of sample (normal *vs*. GBM) probability is higher. The original histological type of the samples is indicated above the graph.

In the training set, the PAM analysis identified 18 cytokines (out of 48 cytokines) that could discriminate GBM sera from normal sera at a threshold value of 1.2 with an error of 4.60% **(S2 Fig)**. The levels of these 18 cytokines were found to be different in the sera of normal controls and GBM patients **(Fig 2A and Table 1)**. We then performed PCA of these 18 cytokines in the training set and found that first three principal components were able to distinguish the normal and GBM sera into two distinct groups **(Fig 2B).** Prediction accuracy estimation by 10-fold cross-validation using PAM revealed that among 13 normal samples, the cytokine signature predicted 11 samples correctly as normal (cross-validated probability more than 0.5) with an error of 15.30% (**Fig 2C; Table 2**). Similarly among 74 GBM samples analyzed, the signature predicted 72 samples correctly as GBM with an error of 2.70% (**Fig 2C; Table 2**). Thus the 18-cytokine signature could discriminate GBM sera from normal sera with an overall diagnostic accuracy of 95.40% (**Table 2**). The sensitivity of the signature for normal sera is 84.61%, whereas for GBM sera, it is 97.30%; the specificity for normal sera is 97.30%, whereas for GBM sera, it is 84.61% (**Table 2**). The robustness of the 18-cytokine signature was also validated internally in the training set by two methods. In random subset analysis, the accuracy of classification by 18-cytokine signature was tested in a subset of 44 samples (normal; n = 7 and GBM; n = 37) derived from training set. It was found that 18-cytokine signature differentiated GBM sera from normal sera with an overall accuracy of 95.45%, sensitivity of 94.87% and specificity of 100.00%. In ***S***upport ***V***ector ***M***achine (SVM) method, training data set was divided into five random equal data sets and then further divided into training and test sets in which the accuracy of classification by 18-cytokine signature was analyzed. SVM based internal validation gave an overall accuracy of 97.77%, sensitivity of 86.67% and specificity of 100.00%.

**Table 1:** Levels of 18 discriminatory cytokines in normal and GBM sera of the training set.

S. No.	Symbol	^a^Serum cytokine expression by bead array					^b^Cytokine expression from TCGA		
		^#^Abundance	p-value	Normal log 2 ratio (Median±SEM)	GBM log 2 ratio (Median±SEM)	Log 2 fold change	Regulation	Log 2 fold change	p-value
1	IL10	High	0.000001	0.4146±0.51	3.6185±0.19	3.89	Up	0.5	0.007705
2	IL17	High	0.000034	-1.5020±0.55	4.6636±0.25	3.87	Down	-0.35	0.000024
3	IL15	High	0.000001	-0.1467±0.15	2.1752±0.25	3.2	Up	1.18	0.000200
4	MIP1α	High	0.004207	-1.4471±0.78	2.7901±0.39	3.19	NS	NS	NS
5	IL2	High	0.002464	-1.5205±0.87	3.4403±0.27	2.86	Up	0.29	0.013905
6	LIF	High	0.000488	0.0000±0.00	3.2854±0.23	2.44	Up	0.71	0.003247
7	TNFα	High	0.006705	-2.1977±0.83	2.7527±0.27	2.29	NS	NS	NS
8	IL6	High	0.000993	-0.2391±0.22	1.3805±0.32	2.03	NS	NS	NS
9	FGF-basic	High	0.002464	-0.4301±0.43	2.1395±0.20	1.98	Up	0.73	0.001428
10	IL4	High	0.00004	-0.0939±0.12	2.1461±0.15	1.77	Up	0.86	0.000024
11	GM-CSF	High	0.047189	0.0000±0.00	2.3384±0.31	1.67	Up	0.5	0.009749
12	IFNγ	High	0.00036	-0.4089±0.30	1.6778±0.15	1.62	Up	0.48	0.004681
13	IL7	High	0.000177	-0.1018±0.16	0.9915±0.12	1.15	Up	2.39	0.000008
14	IL1Rα	High	0.023915	-0.1398±0.16	0.8578±0.18	1.02	Up	0.44	0.044092
15	SCGFβ	High	0.002464	0.0981±0.15	0.7467±0.08	0.68	Up	0.52	0.001428
16	IL12 (P40)	NS	0.26486	1.1393±0.72	0.5251±0.41	-1.15	Down	-0.32	0.001131
17	βNGF	Low	0.000612	0.0987±0.13	-2.1473±0.23	-2.19	Down	-0.29	0.001246
18	IL3	NS	0.214787	0.2316±0.34	-1.2070±0.43	-2.22	NS	NS	NS

^**a**^Cytokine profiling (n = 48) was carried out using bead array method. The levels of 18 discriminatory cytokines of the training set was converted to differential log 2 ratio by dividing the individual sample value with mean of all normal samples for a given cytokine. In this table, median of differential log 2 ratio for a given cytokine and standard error are shown.

^#^The abundance of cytokines in GBM sera when compared to normal sera. “High” refers to cytokine present in elevated levels and “Low” refers to cytokine present in lower levels in GBM sera when compared to normal sera.

^**b**^The TCGA microarray data which is publically available was used to check the transcript levels of 18 differentially abundant cytokines. Non-parametric t-test was conducted with FDR correction using log 2 ratio of normal brain tissue and GBM tumor tissue to identify significant differentially regulated cytokines at transcript level. The regulation, p value and log 2 fold change are provided in the table. “Up” refers to up-regulated and “Down” refers to down-regulated in GBM when compared to normal brain.

NS refers to non-significant.

**Table 2:** The diagnostic accuracy, sensitivity and specificity of 18-cytokine signature in different sample sets.

Samples	Overall Accuracy (%)	^a^Sensitivity (%)		^b^Specificity (%)		Overall error (%)	Normal error (%)	Glioma error (%)
		Normal	Glioma	Normal	Glioma			
Training set (Normal vs. GBM)	95.40 (83/87)	84.61 (11/13)	97.30 (72/74)	97.30 (72/74)	84.61 (11/13)	4.60	15.30	2.70
Test set (Normal vs. GBM)	96.55 (84/87)	92.31 (12/13)	97.30 (72/74)	97.30 (72/74)	92.31 (12/13)	3.40	7.60	2.70
Combined set (Normal vs. GBM)	93.67 (163/174)	80.77 (21/26)	95.95 (142/148)	95.95 (142/148)	80.77 (21/26)	6.30	19.20	4.00
Normal vs. DA	96.00 (48/50)	92.31 (24/26)	100.00 (24/24)	100.00 (24/24)	92.31 (24/26)	4.00	7.00	0.00
Normal vs. AA	95.83 (46/48)	96.15 (25/26)	95.45 (21/22)	95.45 (21/22)	96.15 (25/26)	4.20	3.00	4.00

^**a**^Sensitivity = (the number of positive samples predicted)/(the number of true positives)

^**b**^Specificity = (the number of negative samples predicted)/(the number of true negatives)

**Note:** Values within the parentheses represents (number of samples predicted correctly)/(total number of samples)

### Validation of 18-cytokine signature to distinguish GBM sera from normal sera in the test set

**Fig 3:**
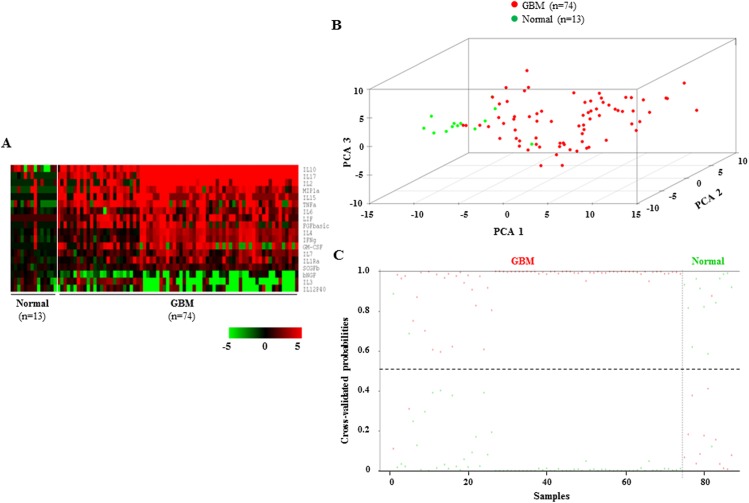
Serum cytokine levels, PCA and cross validated probabilities in test set. **A.** Heat map of supervised one-way hierarchical clustering of 18 PAM-identified cytokines in normal and GBM sera of the test set. A dual-color code was used, with red and green indicating high and low abundance, respectively. The white line separates normal from GBM samples. **B.** PCA was performed using serum levels of 18 PAM-identified cytokines of normal and GBM sera in the test set. A scatter plot was generated using first three principal components for each sample. The color code of the samples is as indicated. **C.** The graph shows detailed probabilities of 10-fold cross-validation for the samples of test set based on the serum levels of 18 PAM-identified cytokines. The probability of a given sample as normal (green color) and GBM (red color) are shown. This was predicted by the PAM program, based on which type of sample (normal *vs*. GBM) probability is higher. The original histological type of the samples is indicated above the graph.

In the test set, the levels of 18 discriminatory cytokines were found to be different between the sera of normal and GBM (**Fig 3A; S3 Table**). PCA analysis revealed that the 18 cytokines (as identified from training set) was able to distinguish normal sera from GBM sera in the test set **(Fig 3B)**. The prediction accuracy estimation by 10-fold cross-validation using PAM revealed that among 13 normal samples, 18-cytokine signature predicted 12 samples correctly as normal with an error of 7.60% (**Fig 3C; Table 2**). Similarly, among 74 GBM samples, our signature predicted 72 samples correctly as GBM with an error of 2.70% (**Fig 3C; Table 2)**. Thus the 18-cytokine signature could discriminate GBM from normal sera with an overall diagnostic accuracy of 96.55% in the test set (**Table 2**). The sensitivity for normal sera is 92.31%, whereas for GBM, it is 97.30%; the specificity for normal is 97.30%, whereas for GBM, it is 92.31% (**Table 2**). We also tested the ability of 18-cytokine signature to discriminate GBM sera from normal sera in the combined set which included both training and test set (normal, n = 26 and GBM, n = 148). The levels of 18 discriminatory cytokines were found to be different between the sera of normal and GBM (**S3A Fig**). PCA analysis revealed that the 18-cytokine signature was able to distinguish normal sera from GBM sera in the combined set (**S3B Fig**). The prediction accuracy estimation by 10-fold cross-validation using PAM revealed that among 26 normal samples, 18-cytokine signature predicted 21 samples correctly as normal with an error of 19.20% **(S3C Fig; Table 2)**. Similarly, among 148 GBM samples, our signature predicted 142 samples correctly as GBM with an error of 4.00% **(S3C Fig; Table 2)**. Next, we tested the ability of 18-cytokine signature to discriminate GBM sera from normal sera by using ***R***eceiver ***O***perating ***C***haracteristic (ROC) curve analysis. While all cytokines individually, except IL3, gave significant area-under-curve (AUC) values, a combined panel containing all 18 cytokines gave an AUC of 1 (**S4 Fig**).

### 18-cytokine signature also discriminates lower grade glioma sera from normal sera

Next, to develop grade-specific cytokine signature, we carried out PCA analysis of 48 cytokine profiles of all samples that belong to DA, AA, GBM and normal sera. Interestingly, we found that DA, AA and GBM samples formed one cluster as against an independent cluster formed by normal sera (**S5A Fig**). Further, PAM analysis between DA *vs*. GBM and AA *vs*. GBM also revealed that none of the 48 cytokines could distinguish GBM sera from DA and AA sera (**S5B and S5C Fig respectively**). These results suggest that the profile of 48 cytokines are similar among DA, AA and GBM, which raises the possibility that 18-cytokine signature developed for GBM sera discrimination can also distinguish DA and AA serum from normal individual serum. Indeed, we found the levels of 18 cytokines to be significantly different in DA and AA sera as against normal sera (**S6A Fig and S7A Fig; S4 Table and S5 Table**). PCA and prediction accuracy estimation by 10-fold cross-validation using PAM revealed that 18-cytokine signature could discriminate DA sera and AA sera from normal sera with an overall accuracy of 96.00% and 95.83% respectively (**S6B, S6C, S7B and S7C Figs; Table 2).**

### Biological significance of the 18-cytokine signature

Next, we investigated the biological significance of the 18-cytokine signature. We found that the transcript levels of 13 cytokines (out of 18) were similarly regulated in GBM tumors (**Table 1**) suggesting that tumor may be the probable source of their differential abundance in the GBM sera. This also suggests that these 13 cytokines by the virtue of their differential regulation in GBM are likely to regulate certain pathways thus contributing to the glioma biology. ***K***yoto ***E***ncyclopedia of ***G***enes and ***G***enomes (KEGG) pathway analysis using 18 cytokines of the signature as input resulted in the enrichment of several pathways (**S8A Fig**). Out of these pathways, two pathways—cytokine-cytokine receptor interaction and JAK-STAT pathway were enriched with maximum significance and more input cytokines (**S8A Fig**). We also carried out univariate and multivariate cox regression survival analysis to know the prognostic value of serum levels of 18 cytokines. Interestingly, we found that only two cytokines–IL17 and IL4 are independent good prognostic indicators (**S8B Fig**). These two cytokines having a good prognostic value in GBM correlates well with the fact that their levels are higher in lower grades, which usually survive longer than GBM (**S8C Fig**).

## Discussion

In the past decade, the study of stromal components and host immune response raised our level of understanding of the development and progression of many tumors to higher levels [29]. Cytokines are important mediators of tumor-stroma interaction, thus facilitating tumor progression and aggressiveness [30]. The purpose of this study was to examine the cytokine alterations in the sera of different grades of glioma and normal healthy individuals and also identify serum based signature for glioma discrimination. We used a specific set of 48 cytokines which are very well studied and implicated in various pathological conditions and cancers including glioma [24,26,31,32,33,34,35,36]. Although there are few serum biomarkers with different utility in GBM [21], currently there are no serum based signature available for GBM. To our knowledge, this study presents a first broad multi-marker screening of serum cytokines in glioma and identified 18-cytokine signature for glioma sera discrimination.

Several serum biomarkers-YKL40, MMP9, B-chain of α_2_-Heremans-Schmid glycoprotein (AHSG), Haptaglobin α2, Osteopontin and IGFBP2 have been reported to identify glioma [19,20,37,38,39,40]. An important limitation in using these markers for glioma diagnosis is that a subset of GBM patients has serum levels of these proteins similar to that of normal healthy individuals which reduces the accuracy of discrimination. While each of these markers represents unique altered pathway in the tumors, combining the markers has advantages over individual markers and will provide more accurate information in clinical settings. Thus, a multi-marker panel identified in this study is likely to work efficiently and help in accurate diagnosis.

At present, imaging by MRI or CT scan of brain is the primary investigation for evaluation of patient with a suspicion of brain tumor, which will likely remain the mainstay for screening. However, tissue biopsy by a neurosurgical procedure is mandatory for further clinical management. The serum cytokine signature can be very useful in identifying glioma in a patient with brain parenchymal lesion, thereby avoiding a need for the biopsy. An evaluation of this signature among various brain parenchymal lesions, both neoplastic and non-neoplastic will further strengthen the utility of this signature. Moreover, we propose that this cytokine signature can be used to monitor the treatment response and tumor recurrence, which may be more cost effective than performing periodical CT scans. It is possible that the imaging can be considered in patients of glioma when they demonstrate an increase in the levels of identified cytokines.

The 18-cytokine signature identified in this study comprises 15 cytokines to be present in elevated amounts while remaining three in lower amounts in GBM when compared to normal serum samples. Among this 18-cytokines, 13 of them exhibited similar regulation at transcript levels in the GBM tissue as well. We found 11 of them to be up-regulated (IL10, IL15, IL2, LIF, FGF basic, IL4, GM-CSF, IFNγ, IL7, IL1RA and SCGFβ) while 2 to be down-regulated (IL12 (P40) and βNGF) in GBM tissue when compared to normal brain samples. Literature survey confirmed the differential regulation of many of the cytokines and also provided potential functions during glioma development and therapy. For example, IL10, LIF and IL1RA were similarly regulated in GBM tumor at transcript level as measured by RNase protection assay [25]. IL10, an upregulated cytokine in our study, was shown to be expressed in glioma by few studies and functions to inhibit antitumor response while promoting proliferation and motility [41,42,43,44]. IL2 has been used for therapy in glioma which increased the patient’s survival [45]. LIF has been reported to play an essential role in inducing the self-renewal capacity of glioma-initiating cells and thus promoting oncogenesis [46]. FGF basic is expressed in glioma and it promotes glioma growth and angiogenesis [47,48,49]. IL4 stimulation causes Stat3 activation in GBM and modulates Bcl-2 family of antiapoptotic proteins thereby contributing to pathogenesis of GBM [50]. IL4R is found in abundance in glioma tumor cells and hence IL4 along with *Pseudomonas* exotoxin is used as immunotoxin therapy in clinical trials [51]. GM-CSF was used as an adjuvant in vaccination along with whole tumor lysate. Although clinical improvement was observed, a subset of patient displayed delayed-type hypersensitivity [52]. Several studies have used IFN in glioma therapy [53,54,55]. IFNγ inhibits proliferation, migration and angiogenesis in glioma [56,57,58]. Intra-tumoral IFNγ treatment in a randomized trial did not show significant difference in tumor progression and median survival of patients [59]. However, other studies wherein IFNγ in combination with other agents showed anti-tumor response [60,61]. IL7 expression in glioma cell lines and tumor samples positively correlated to the extent of chemoresistance to cisplatin and thereby suggesting its importance in clinical management of glioma [62]. IL1RA is expressed by some glioma cell lines and GBM tumors and supports growth in autocrine fashion [63,64]. More recently, IL1RA conjugated to super-paramagnetic iron oxide nanoparticles was used to target glioma in experimental models [65]. IL12 found to be down-regulated in our study is an inhibitor of angiogenesis and display anti-tumorigenic function [66]. βNGF was found to inhibit growth of rat C6 glioma cells [67].

The pathway analysis using 18 cytokines of the signature enriched cytokine-cytokine receptor interaction and JAK-STAT pathway with highest significance. Cytokines mainly function through their receptors with the involvement of members of the JAK-STAT pathway [68,69]. These pathways are demonstrated to be activated and have an importance in the glioma pathogenesis [70,71]. Thus the cytokines which are present in differential concentrations in glioma sera are also likely to participate in glioma development and progression.

In conclusion, our study identified an 18-cytokine signature that can distinguish glioma sera from normal sera with high accuracy. These findings suggest that the serum cytokine signature can be very useful in identifying glioma in a patient with brain parenchymal lesion, thereby avoiding a need for the biopsy and also in monitoring treatment response and recurrence.

## Supporting Information

S1 Fig Statistical analysis of age and gender distribution among the samples used in this study. The significance of distribution was analyzed by non-parametric t-test using Graph Pad Prism (version 5.01) and the p values are indicated.
(TIF)

S2 Fig PAM analysis to identify serum cytokine signature using training set. **A.** Plot showing mis-classification error for the 48 input cytokines from PAM analysis in the training set. The broken line indicate threshold value of 1.2 corresponding to 18 cytokines (15 up-regulated and 3 down-regulated) which classified normal (green; n = 13) and GBM (red; n = 74) samples with classification error of 4.60%. **B.** Tabulated PAM output using training set.
(TIF)

S3 Fig Serum cytokine levels, PCA and cross validated probabilities in the combined set. **A**. Heat map of supervised one-way hierarchical clustering of 18 PAM-identified cytokines in normal (n = 26) and GBM (n = 148) sera in the combined set. A dual-color code was used, with red and green indicating high and low abundance, respectively. The white line separates normal from GBM samples. **B.** PCA was performed using serum levels of 18 PAM-identified cytokines of normal and GBM sera in the combined set. A scatter plot was generated using first three principal components for each sample. The color code of the samples is as indicated. **C.** The graph shows detailed probabilities of 10-fold cross-validation for the samples of combined set based on the serum levels of 18 PAM-identified cytokines. The probability of a given sample as normal (green color) and GBM (red color) are shown. This was predicted by the PAM program, based on which type of sample (normal *vs*. GBM) probability is higher. The original histological type of the samples is indicated above the graph.
(TIF)

S4 Fig ROC curve analysis using 18 cytokines of the signature. **A.** Graphical representation of ROC curve analysis output using 18 cytokines of the signature. Color code is as indicated. **B.** AUC values for discriminating GBM from normal controls using 18 cytokines individually and as a combined panel and corresponding p values are shown in the tabular form.
(TIF)

S5 Fig PCA and PAM using serum levels of 48 cytokines. **A.** PCA was performed using serum levels of 48 cytokines of normal (n = 26), DA (n = 24), AA (n = 22), and GBM (n = 148) samples. A scatter plot was generated using first three principal components for each sample. The color code of the samples is as indicated. **B and C.** Plot showing mis-classification error from PAM analysis using serum levels of 48 cytokines for DA *vs*. GBM and AA *vs*. GBM respectively.
(TIF)

S6 Fig Serum levels of 18 cytokines, PCA and cross validated probabilities for normal *vs*. DA samples. **A**. Heat map of supervised one-way hierarchical clustering of 18 PAM-identified cytokines in normal (n = 26) and DA (n = 24) sera. A dual-color code was used, with red and green indicating high and low abundance, respectively. The white line separates normal from DA samples. **B.** PCA was performed using serum levels of 18 PAM-identified cytokines in normal and DA samples. A scatter plot was generated using first three principal components for each sample. The color code of the samples is as indicated. **C.** The graph shows detailed probabilities of 10-fold cross-validation for the samples based on the serum levels of 18 PAM-identified cytokines. The probability of a given sample as normal (green color) and DA (red color) are shown. This was predicted by the PAM program, based on which type of sample (normal *vs*. DA) probability is higher. The original histological type of the samples is indicated above the graph.
(TIF)

S7 Fig Serum levels of 18 cytokines, PCA and cross validated probabilities for normal *vs*. AA samples. **A**. Heat map of supervised one-way hierarchical clustering of 18 PAM-identified cytokines in normal (n = 26) and AA (n = 22) sera. A dual-color code was used, with red and green indicating high and low abundance, respectively. The white line separates normal from AA samples. **B.** PCA was performed using serum levels of 18 PAM-identified cytokines in normal and AA samples. A scatter plot was generated using first three principal components for each sample. The color code of the samples is as indicated. **C.** The graph shows detailed probabilities of 10-fold cross-validation for the samples based on the serum levels of 18 PAM-identified cytokines. The probability of a given sample as normal (green color) and AA (red color) are shown. This was predicted by the PAM program, based on which type of sample (normal *vs*. AA) probability is higher. The original histological type of the samples is indicated above the graph.
(TIF)

S8 Fig Pathway analysis and survival analysis using 18 cytokines of the signature. **A.** Tabulated KEGG pathway analysis output using 18 cytokines of the signature is shown. **B.** Cox proportional hazard ratio analysis using serum levels of 18 cytokines of the signature and clinical data from 96 GBM patients. **C.** Scatter plot representation of serum levels of IL17 and IL4 in normal (n = 26), DA (n = 24), AA (n = 22), and GBM (n = 148) samples. Statistical analysis (one-way ANOVA) was performed and the p values are indicated. The horizontal line represents mean value, ns = non-significant.
(TIF)

S1 Table Clinical data of the samples used in this study. (XLSX)

S2 Table List of 48 cytokines profiled in this study. (DOCX)

S3 Table Levels of 18 discriminatory cytokines in normal and GBM sera of the test set. (DOCX)

S4 Table Levels of 18 discriminatory cytokines in normal and DA sera. (DOCX)

S5 Table Levels of 18 discriminatory cytokines in normal and AA sera. (DOCX)

## Abbreviations

GBM: Glioblastoma
PAM: Prediction Analysis for Microarrays
PCA: Principal Component Analysis
