# Supplementary figures and images for: An Eighteen Serum Cytokine Signature for Discriminating Glioma from Normal Healthy Individuals
Source: PLoS One. 2015 Sep 21;10(9):e0137524. doi: 10.1371/journal.pone.0137524 (PMC4577083; doi:10.1371/journal.pone.0137524)

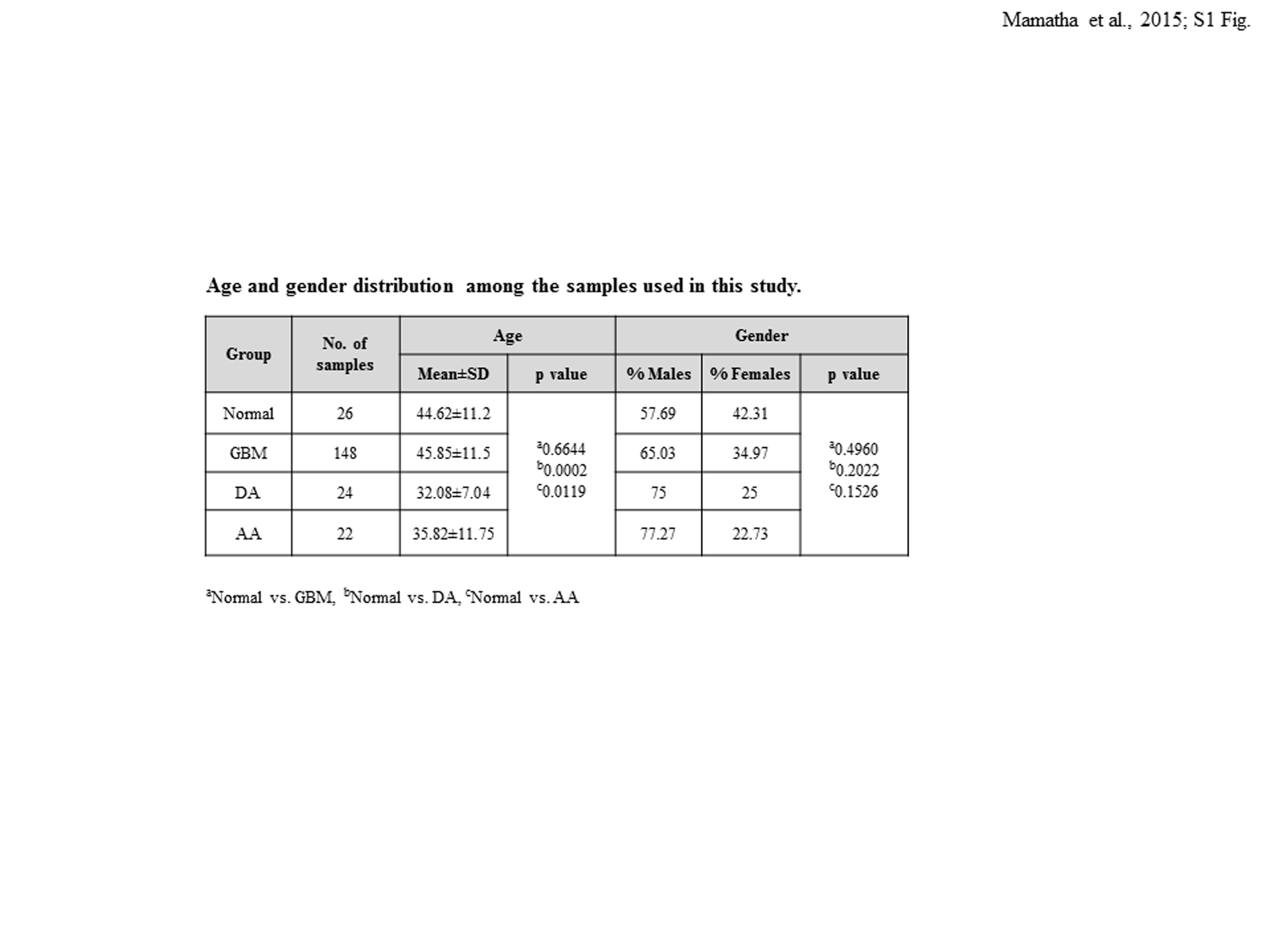

Supplement: S1 Fig — The significance of distribution was analyzed by non-parametric t-test using Graph Pad Prism (version 5.01) and the p values are indicated. (TIF) [file pone.0137524.s001.tif]

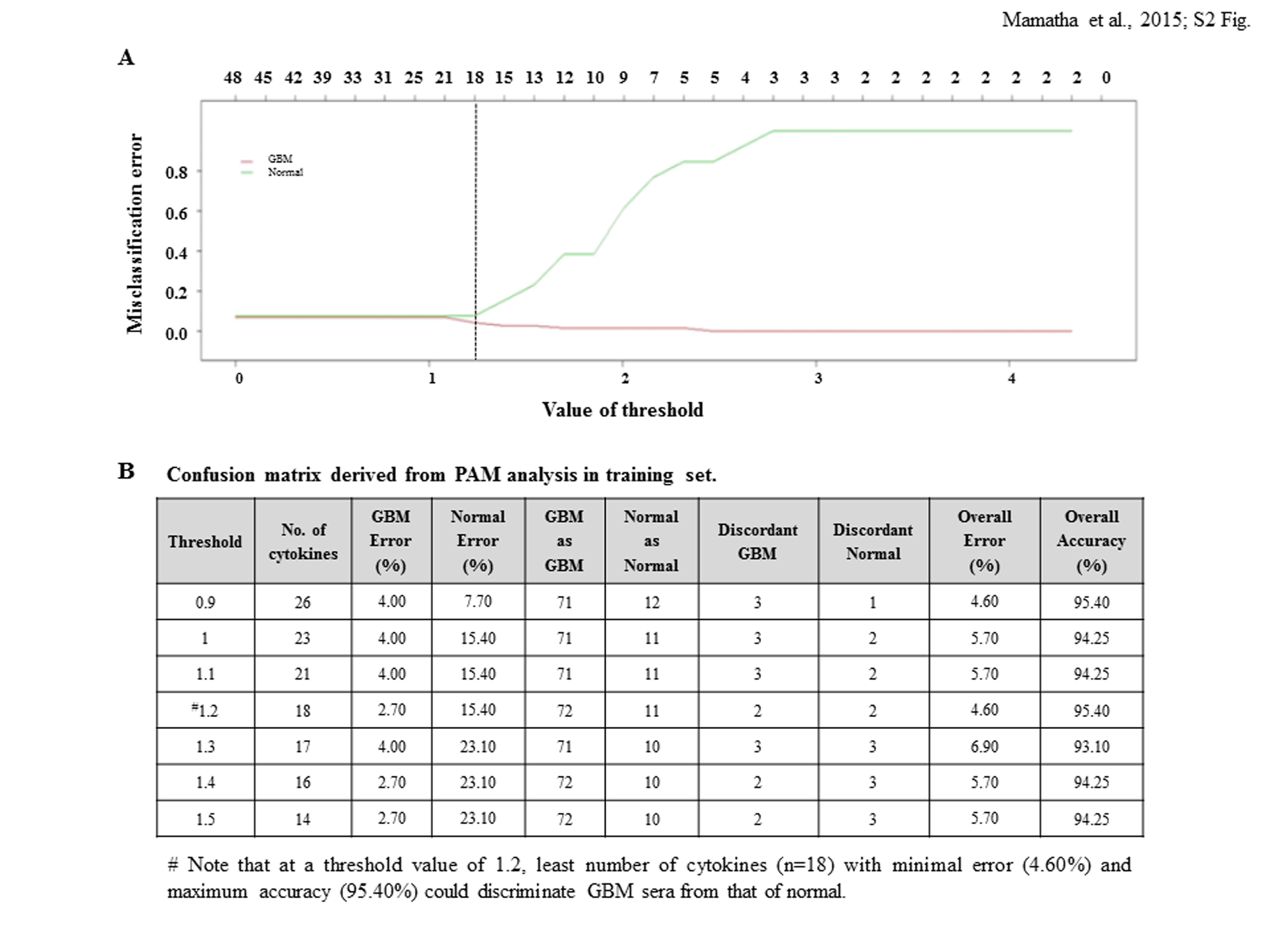

Supplement: S2 Fig — A. Plot showing mis-classification error for the 48 input cytokines from PAM analysis in the training set. The broken line indicate threshold value of 1.2 corresponding to 18 cytokines (15 up-regulated and 3 down-regulated) which classified normal (green; n = 13) and GBM (red; n = 74) samples with classification error of 4.60%. B. Tabulated PAM output using training set. (TIF) [file pone.0137524.s002.tif]

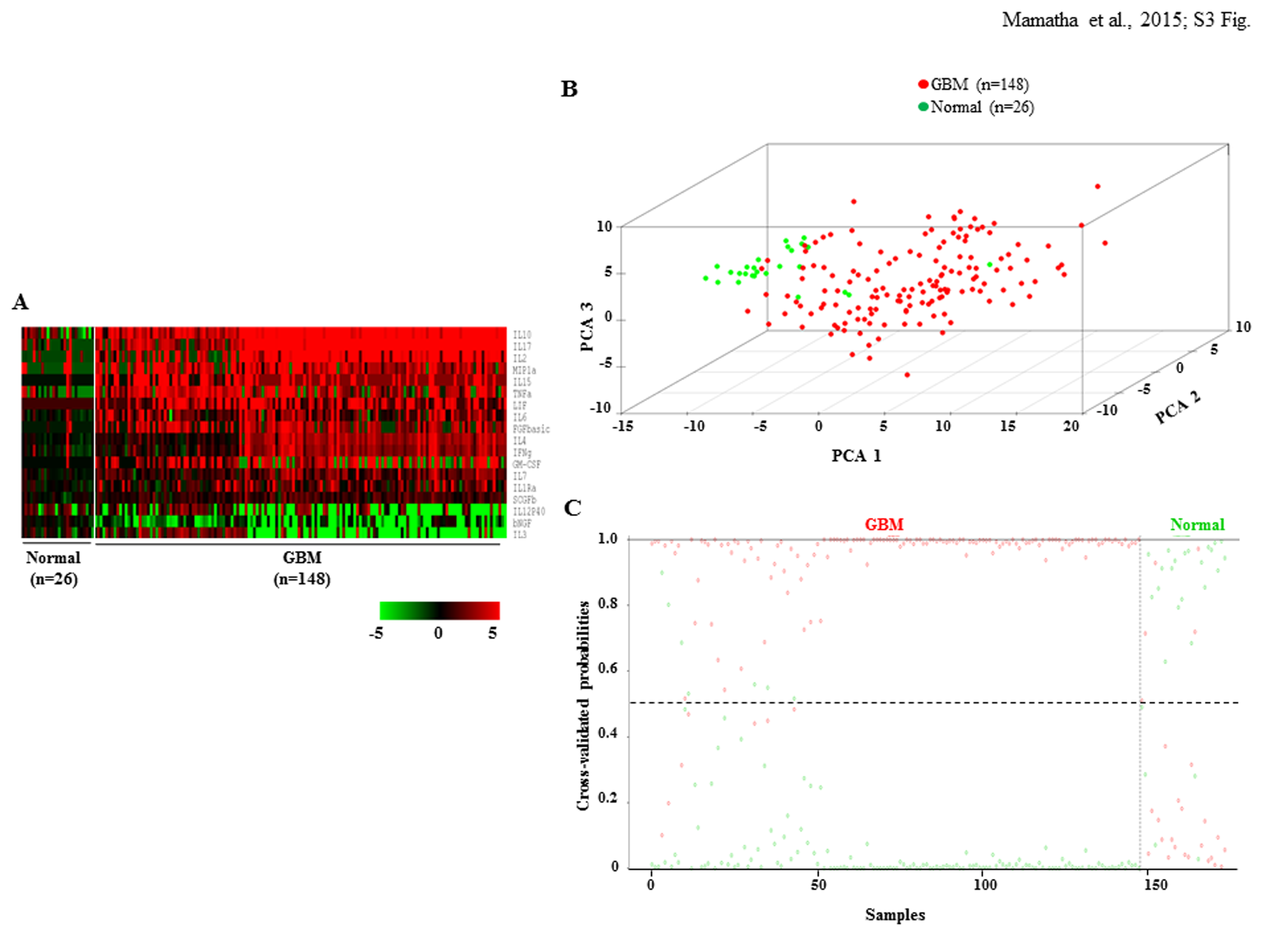

Supplement: S3 Fig — A. Heat map of supervised one-way hierarchical clustering of 18 PAM-identified cytokines in normal (n = 26) and GBM (n = 148) sera in the combined set. A dual-color code was used, with red and green indicating high and low abundance, respectively. The white line separates normal from GBM samples. B. PCA was performed using serum levels of 18 PAM-identified cytokines of normal and GBM sera in the combined set. A scatter plot was generated using first three principal components for each sample. The color code of the samples is as indicated. C. The graph shows detailed probabilities of 10-fold cross-validation for the samples of combined set based on the serum levels of 18 PAM-identified cytokines. The probability of a given sample as normal (green color) and GBM (red color) are shown. This was predicted by the PAM program, based on which type of sample (normal vs. GBM) probability is higher. The original histological type of the samples is indicated above the graph. (TIF) [file pone.0137524.s003.tif]

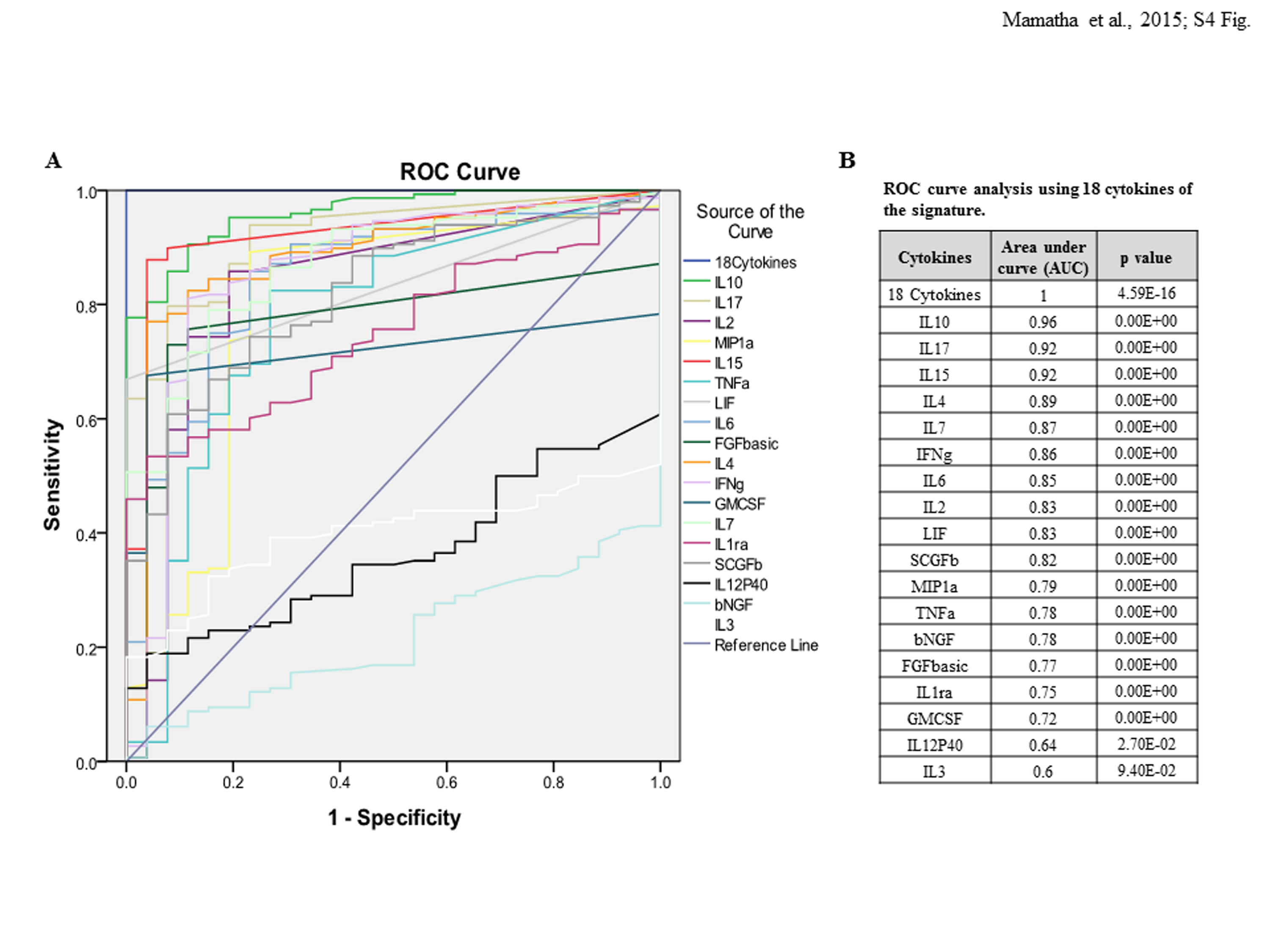

Supplement: S4 Fig — A. Graphical representation of ROC curve analysis output using 18 cytokines of the signature. Color code is as indicated. B. AUC values for discriminating GBM from normal controls using 18 cytokines individually and as a combined panel and corresponding p values are shown in the tabular form. (TIF) [file pone.0137524.s004.tif]

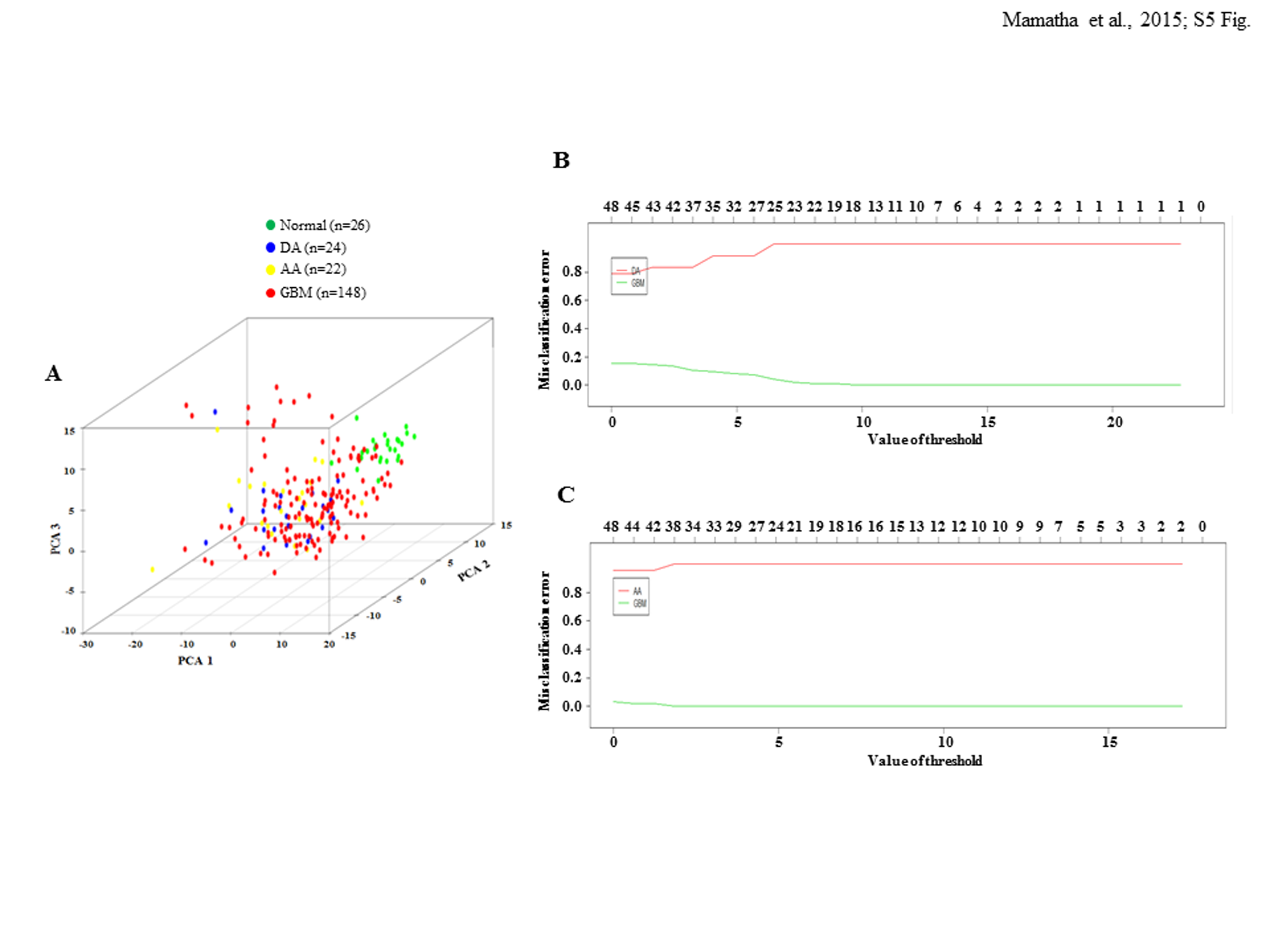

Supplement: S5 Fig — A. PCA was performed using serum levels of 48 cytokines of normal (n = 26), DA (n = 24), AA (n = 22), and GBM (n = 148) samples. A scatter plot was generated using first three principal components for each sample. The color code of the samples is as indicated. B and C. Plot showing mis-classification error from PAM analysis using serum levels of 48 cytokines for DA vs. GBM and AA vs. GBM respectively. (TIF) [file pone.0137524.s005.tif]

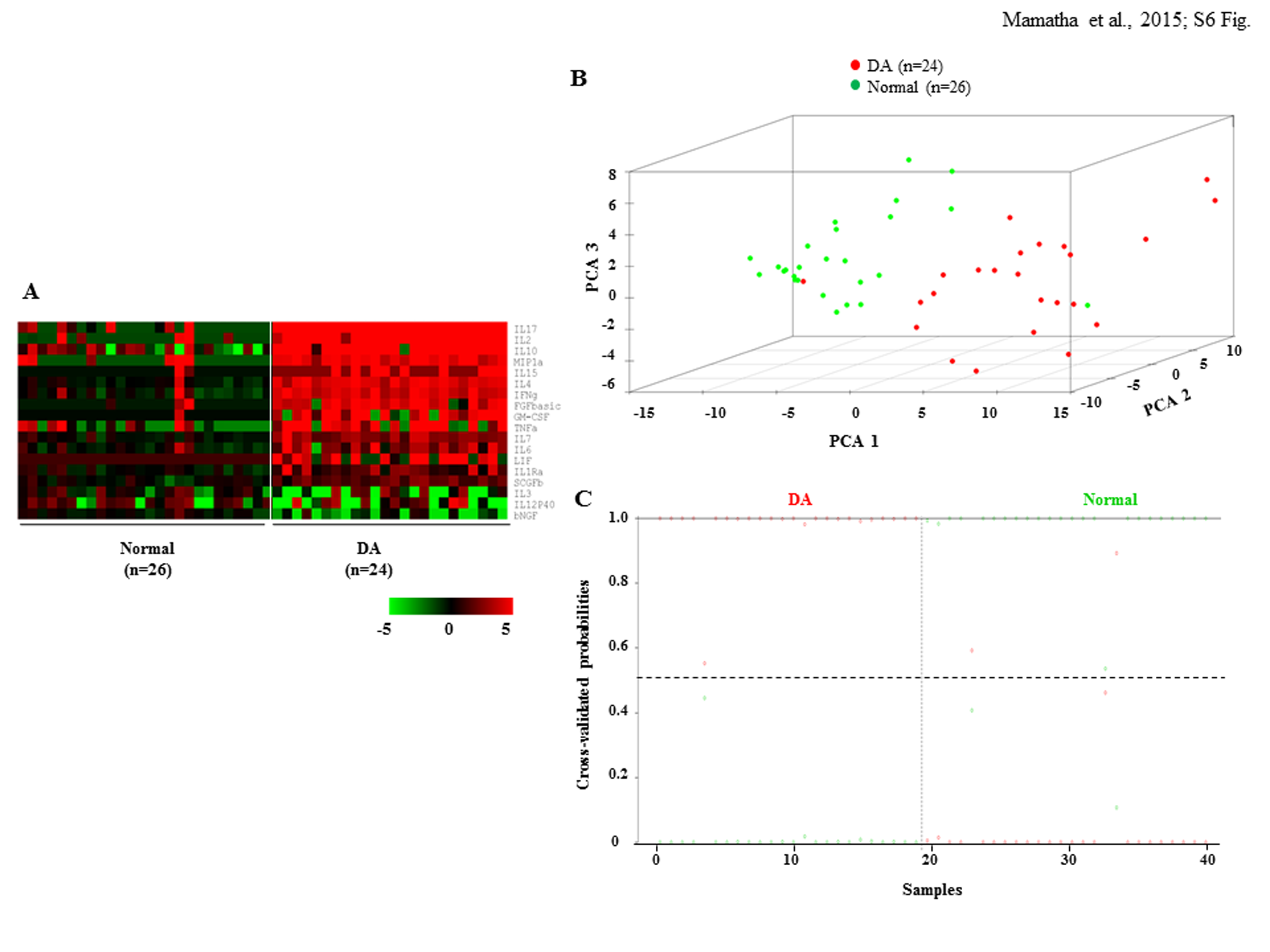

Supplement: S6 Fig — A. Heat map of supervised one-way hierarchical clustering of 18 PAM-identified cytokines in normal (n = 26) and DA (n = 24) sera. A dual-color code was used, with red and green indicating high and low abundance, respectively. The white line separates normal from DA samples. B. PCA was performed using serum levels of 18 PAM-identified cytokines in normal and DA samples. A scatter plot was generated using first three principal components for each sample. The color code of the samples is as indicated. C. The graph shows detailed probabilities of 10-fold cross-validation for the samples based on the serum levels of 18 PAM-identified cytokines. The probability of a given sample as normal (green color) and DA (red color) are shown. This was predicted by the PAM program, based on which type of sample (normal vs. DA) probability is higher. The original histological type of the samples is indicated above the graph. (TIF) [file pone.0137524.s006.tif]

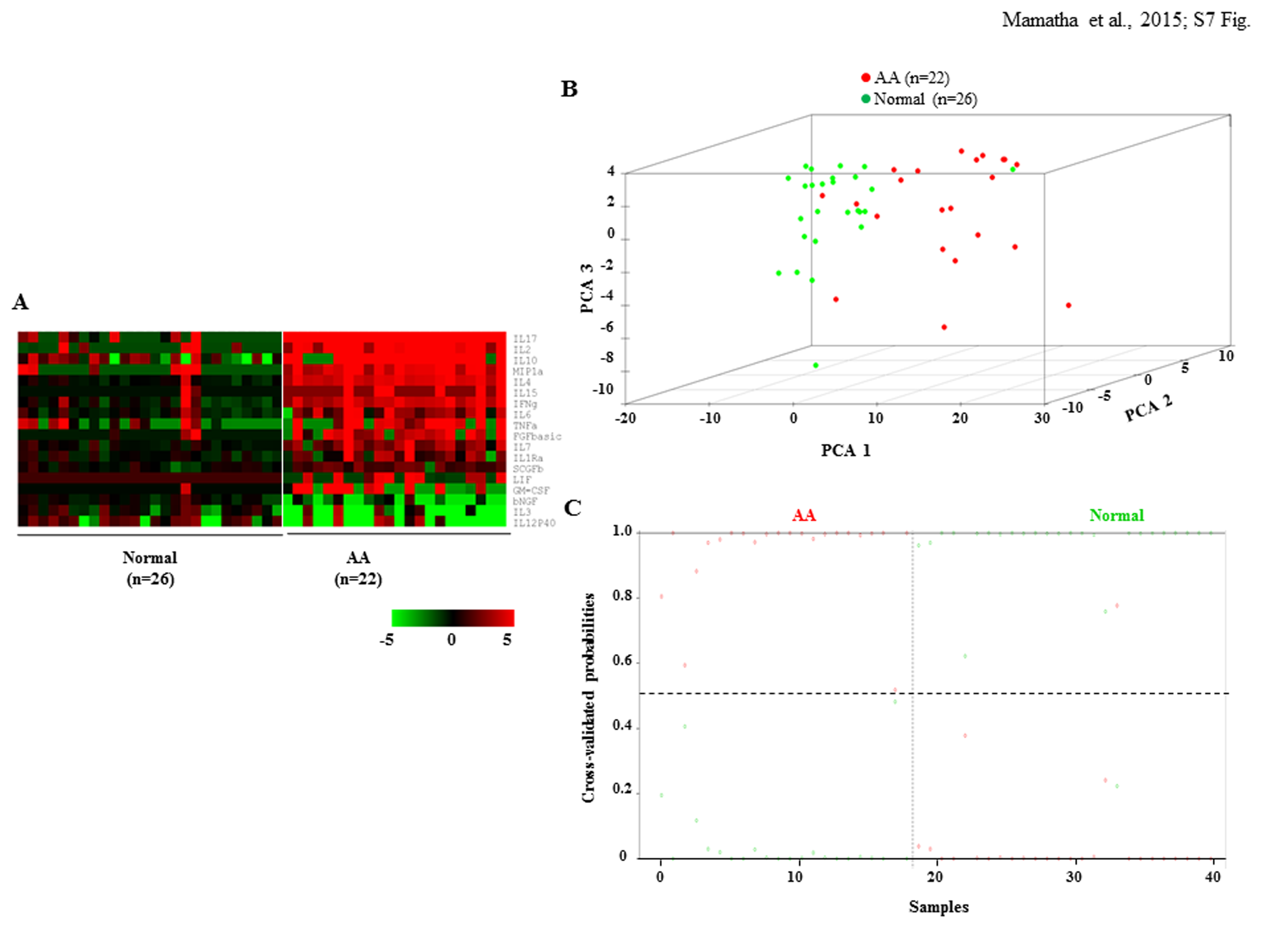

Supplement: S7 Fig — A. Heat map of supervised one-way hierarchical clustering of 18 PAM-identified cytokines in normal (n = 26) and AA (n = 22) sera. A dual-color code was used, with red and green indicating high and low abundance, respectively. The white line separates normal from AA samples. B. PCA was performed using serum levels of 18 PAM-identified cytokines in normal and AA samples. A scatter plot was generated using first three principal components for each sample. The color code of the samples is as indicated. C. The graph shows detailed probabilities of 10-fold cross-validation for the samples based on the serum levels of 18 PAM-identified cytokines. The probability of a given sample as normal (green color) and AA (red color) are shown. This was predicted by the PAM program, based on which type of sample (normal vs. AA) probability is higher. The original histological type of the samples is indicated above the graph. (TIF) [file pone.0137524.s007.tif]

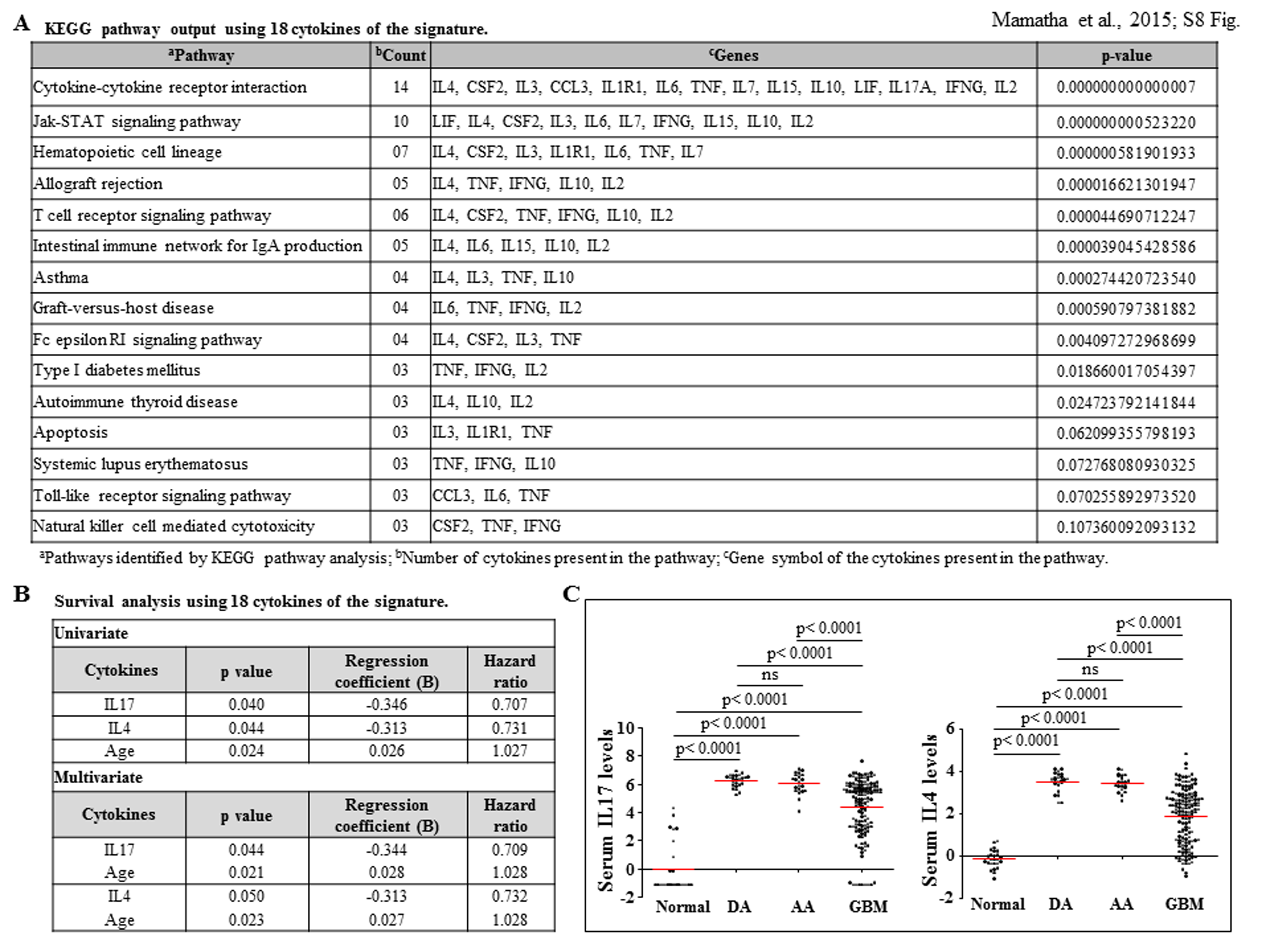

Supplement: S8 Fig — A. Tabulated KEGG pathway analysis output using 18 cytokines of the signature is shown. B. Cox proportional hazard ratio analysis using serum levels of 18 cytokines of the signature and clinical data from 96 GBM patients. C. Scatter plot representation of serum levels of IL17 and IL4 in normal (n = 26), DA (n = 24), AA (n = 22), and GBM (n = 148) samples. Statistical analysis (one-way ANOVA) was performed and the p values are indicated. The horizontal line represents mean value, ns = non-significant. (TIF) [file pone.0137524.s008.tif]
