# Supplementary material for: An Eighteen Serum Cytokine Signature for Discriminating Glioma from Normal Healthy Individuals
Source: PLoS One. 2015 Sep 21;10(9):e0137524. doi: 10.1371/journal.pone.0137524 (PMC4577083; doi:10.1371/journal.pone.0137524)
Supplement: S2 Table — (DOCX) [file pone.0137524.s010.docx]

| **S2 Table.** **List of 48 cytokines profiled in this study.** | | | | | |
| --- | --- | --- | --- | --- | --- |
| **27-plex** | | | **21- plex** | | |
| **S.No.** | **Symbol** | **Name** | **S.No** | **Symbol** | **Name** |
| 1 | IL1β | Interleukin 1Beta | 1 | IFNα2 | Interferon Alpha 2 |
| 2 | IL2 | Interleukin 2 | 2 | IL1α | Interleukin 1Alpha |
| 3 | IL1rα | Interleukin 1 Receptor Alpha | 3 | IL2rα | Interleukin 2 Receptor Alpha |
| 4 | IL4 | Interleukin 4 | 4 | IL3 | Interleukin 3 |
| 5 | IL5 | Interleukin 5 | 5 | IL12 (p40) | Interleukin 12 subunit p40 |
| 6 | IL6 | Interleukin 6 | 6 | IL16 | Interleukin 16 |
| 7 | IL7 | Interleukin 7 | 7 | IL18 | Interleukin 18 |
| 8 | IL8 | Interleukin 8 | 8 | CTACK | Cutaneous T-cell Attracting Chemokine |
| 9 | IL9 | Interleukin 9 | 9 | GROα | Growth Regulated Alpha Protein |
| 10 | IL10 | Interleukin 10 | 10 | HGF | Hepatocyte growth factor |
| 11 | IL12 (p70) | Interleukin 12 | 11 | TRAIL | TNF Related Apoptosis Inducing Ligand |
| 12 | IL13 | Interleukin 13 | 12 | LIF | Leukemia Inhibitory Factor |
| 13 | IL15 | Interleukin 15 | 13 | MCP3 | Monocyte Chemoattractant Protein 3 |
| 14 | IL17 | Interleukin 17 | 14 | MCSF | Macrophage Colony Stimulating Factor |
| 15 | Eotaxin | N/A | 15 | MIF | Macrophage Migration Inhibitory Factor |
| 16 | Basic FGF | Basic Fibroblast Growth Factor | 16 | MIG | Monokine Induced by Gamma Interferon |
| 17 | GCSF | Granulocyte Colony Stimulating Factor | 17 | βNGF | Nerve Growth Factor Beta |
| 18 | GMCSF | Granulocyte Macrophage Colony Stimulating Factor | 18 | SCF | Stem Cell Factor |
| 19 | VEGF | Vascular Endothelial Growth Factor A | 19 | SCGFβ | Stem cell growth factor |
| 20 | IFNγ | Interferon Gamma | 20 | SDF1α | Stromal cell Derived Factor 1 |
| 21 | IP10 | Interferon Gamma Inducible Protein 10 | 21 | TNFβ | Tumor Necrosis Factor Beta |
| 22 | MCP1 | Monocyte Chemoattractant Protein 1 |  |  |  |
| 23 | MIP1α | Macrophage Inflammatory Protein 1 Alpha |  |  |  |
| 24 | MIP1β | Macrophage Inflammatory Protein 1 Beta |  |  |  |
| 25 | PDGFBB | Platelet-Derived Growth Factor-BB |  |  |  |
| 26 | RANTES | Regulated upon activation, normally T-expressed, and presumably secreted |  |  |  |
| 27 | TNFα | Tumor Necrosis Factor Alpha |  |  |  |
