# Supplementary material for: An Eighteen Serum Cytokine Signature for Discriminating Glioma from Normal Healthy Individuals
Source: PLoS One. 2015 Sep 21;10(9):e0137524. doi: 10.1371/journal.pone.0137524 (PMC4577083; doi:10.1371/journal.pone.0137524)
Supplement: S4 Table — (DOCX) [file pone.0137524.s012.docx]

| **S4 Table. Levels of 18 discriminatory cytokines in normal and DA sera.** | | | | | | |
| --- | --- | --- | --- | --- | --- | --- |
| **S.No.** | **Symbol** | **Abundance^#^** | **p-value** | **Normal log 2 ratio  Median±SEM^a^** | **DA log 2 ratio Median±SEM^b^** | **Log 2  fold change^c^** |
| 1 | IL10 | High | 0.00000015 | 1.0782±0.56 | 5.3935±0.51 | 5.28 |
| 2 | IL17 | High | 0.00000001 | -1.1322±0.36 | 6.3851±0.09 | 6.24 |
| 3 | IL15 | High | 0.00000001 | -0.2348±0.17 | 2.0871±0.45 | 3.69 |
| 4 | MIP1α | High | 0.00000704 | -1.2496±0.47 | 3.7454±0.35 | 4.18 |
| 5 | IL2 | High | 0.00000012 | -1.0710±0.49 | 5.6440±0.29 | 5.58 |
| 6 | LIF | NS | 0.10747638 | 0.0000±0.00 | 3.2164±0.53 | 2.20 |
| 7 | TNFα | High | 0.00271625 | -2.0246±0.56 | 3.8254±0.66 | 2.82 |
| 8 | IL6 | High | 0.00000704 | -0.3083±0.19 | 2.0670±0.50 | 2.35 |
| 9 | FGFbasic | High | 0.00000312 | -0.3772±0.24 | 3.5147±0.36 | 3.06 |
| 10 | IL4 | High | 0.00000001 | -0.1475±0.16 | 3.6165±0.09 | 3.51 |
| 11 | GM-CSF | High | 0.00038700 | -0.1310±0.13 | 3.4184±0.63 | 2.96 |
| 12 | IFNγ | High | 0.00000008 | -0.4058±0.24 | 3.3681±0.12 | 3.42 |
| 13 | IL7 | High | 0.00000001 | -0.1681±0.11 | 2.1888±0.30 | 2.43 |
| 14 | IL1Rα | High | 0.00001190 | -0.0532±0.12 | 1.1945±0.25 | 1.47 |
| 15 | SCGFβ | High | 0.00000008 | 0.0307±0.12 | 1.3925±0.11 | 1.39 |
| 16 | IL12P40 | NS | 0.19141422 | 0.9465±0.46 | -0.1437±0.76 | -1.43 |
| 17 | βNGF | Low | 0.00093200 | 0.0920±0.12 | -1.9480±0.36 | -1.76 |
| 18 | IL3 | NS | 0.10475239 | 0.1729±0.25 | -0.7119±0.54 | -1.30 |

^#^The abundance of cytokines in DA sera when compared to normal sera. “High” refers to cytokine present in elevated levels and “Low” refers to cytokine present in lower levels in DA sera when compared to normal sera.

**^a^**The levels of 18 discriminatory cytokines in normal samples (n=26) was converted to differential log 2 ratio by dividing the individual sample value with mean of all normal samples for a given cytokine. In this table, median of differential log 2 ratio for a given cytokine and standard error are shown.

**^b^**The levels of 18 discriminatory cytokines in DA samples (n=24) was converted to differential log 2 ratio by dividing the individual sample value with mean of all normal samples for a given cytokine. In this table, median of differential log 2 ratio for a given cytokine and standard error are shown.

**^c^**Log 2 fold change is calculated by subtracting mean of normal differential log 2 ratio from mean of DA differential log 2 ratio.
